# The global burden of osteoarthritis hand: lessons from the Global Burden of Disease Study 1990–2021

**DOI:** 10.3389/fmed.2025.1616132

**Published:** 2025-07-31

**Authors:** Jiyong Wei, Zhongyi Su, Songmu Pan, Zhuan Zou, Gui Liao, Guangxiong Li, Huijiang Liu, Guipeng Lan, Ronghe Gu, Yanni Lan

**Affiliations:** 1 Department of Orthopedic Surgery, The First People's Hospital of Nanning, The Fifth Affiliated Hospital of Guangxi Medical University, Nanning, Guangxi, China; 2 Department of Pharmacy, The People's Hospital of Guangxi Zhuang Autonomous Region & Guangxi Academy of Medical Sciences, Nanning, Guangxi, China; 3 Department of Bone Surgery, The Eight People's Hospital of Nanning, Nanning, Guangxi, China

**Keywords:** osteoarthritis, hand, global, disease burden, GBD

## Abstract

**Background:**

Hand osteoarthritis (HOA), a disabling musculoskeletal disorder, poses a significant global burden but remains understudied relative to other osteoarthritis (OA) subtypes. Using data from the Global Burden of Disease (GBD) Study 1990–2021, this analysis characterizes HOA epidemiology, temporal trends, and future projections.

**Methods:**

GBD 2021 data on HOA incidence, prevalence, and disability-adjusted life-years (DALYs) were stratified by sex, age, Socio-demographic Index (SDI) regions, GBD regions, and countries. Temporal trends (1990–2021) were assessed via estimated annual percentage changes (EAPCs), with future projections (2022–2046) generated using an age-period-cohort (APC) model.

**Results:**

In 2021, HOA accounted for 10.37 million incidence cases, 194.28 million prevalence cases, and 6.17 million DALYs. Female burdens were 1.8–2.0 times higher than males in absolute terms and 1.75–1.78 times higher in age-standardized rates (ASRs). Incidence cases peaked in advanced ages before declining, while prevalence/DALYs rose monotonically with age. Middle SDI regions had the highest absolute burdens, whereas high SDI regions showed the highest ASRs. Central Asia emerged as a burden hotspot, while minimal health system regions and sub-Saharan Africa had the lowest rates. From 1990 to 2021, global incidences increased 142%. Low/middle-income regions (such as South Asia) saw significant increases, contrasting with declines in high-income areas (such as Western Europe). APC projections indicate continued growth through 2046, with male/female incidences rising 69.6%/51.6% and ASRs increasing for both sexes.

**Conclusion:**

HOA represents a growing global challenge with pronounced sex/age/regional disparities. Targeted interventions in high-burden regions, aging populations, and risk factor management are critical to mitigate projected burden increases.

## 1 Introduction

Osteoarthritis (OA) is a leading contributor to chronic pain and disability globally, particularly in the context of aging populations and lifestyle transitions (1). Among its subtypes, hand osteoarthritis (HOA) poses a substantial yet underrecognized burden due to its impact on manual function, productivity, and quality of life. Despite its clinical significance, HOA has historically received less attention than knee or hip OA, even as emerging data from the Global Burden of Disease (GBD) Study 2021 reveal its escalating incidence, prevalence, and disability toll (2).

GBD 2021 estimates suggest that OA affected 595 million individuals worldwide in 2020, with HOA accounting for a considerable share (1, 3). By 2050, HOA cases are projected to rise by 48.6%, largely driven by population aging, increasing obesity, and occupational exposures (1, 4). The age-standardized disability-adjusted life-years (DALYs) rate for OA increased by 9.5% between 1990 and 2020, underscoring its growing global impact (1). While high sociodemographic index (SDI) countries report the highest prevalence, low- and middle-SDI regions are experiencing the most rapid increases in incidence—reflecting healthcare disparities, under diagnosis, and delayed access to treatment (2, 5). For instance, Asia-Pacific countries report disproportionately high HOA burdens, potentially due to genetic predispositions, manual labor intensity, and diagnostic delays (2, 6).

Elevated body mass index remains the dominant modifiable risk factor, implicated in 20.4% of global OA cases, including HOA (1). Yet, occupational stressors—such as repetitive manual tasks in agriculture or manufacturing—and joint trauma are underexplored contributors, particularly in younger working populations (4, 7). A worrying trend is the earlier onset of HOA, with individuals under 55 increasingly affected, leading to prolonged disability, joint surgeries, and estimated annual productivity losses exceeding US$106.8 billion in some economies (8). These challenges are compounded by the lack of disease-modifying treatments, with most interventions aimed solely at symptom relief (1).

The GBD framework has enabled a systematic assessment of HOA's global epidemiology and its regional disparities. Countries such as Denmark demonstrate success in reducing OA rates through proactive public health measures, while low-SDI settings continue to face rising incidence amidst limited resources (2, 5). Meanwhile, gender disparities persist, with women experiencing 20%−30% higher HOA prevalence—largely due to hormonal influences and disproportionate involvement in caregiving and manual occupations (2, 6). Aging and population growth remain key drivers, accounting for over 60% of the rising HOA burden in low-SDI settings (5).

Nonetheless, important knowledge gaps remain. The current GBD framework does not incorporate occupational hazards or joint trauma as formal risk factors, limiting the identification of actionable prevention targets (1, 7). Furthermore, economic data on HOA burden are scarce, particularly in resource-limited settings where out-of-pocket costs contribute to health inequities (9). Integrating socioeconomic and occupational determinants—such as informal labor, ergonomic exposures, and income inequality—into future GBD iterations will be essential to guide effective interventions.

In light of these persisting gaps, the present study provides the first comprehensive assessment of the global, regional, and national burden of HOA from 1990 to 2021, with forecasts extending to 2046. Leveraging data from the GBD 2021 study and employing age-period-cohort (APC) modeling techniques, we delineate temporal trends, sex- and age-specific patterns, and geospatial heterogeneity in HOA burden. This work identifies emerging hotspots and divergent trajectories across sociodemographic contexts, offering critical evidence to guide equity-focused musculoskeletal health strategies and inform policy prioritization in the post-2020 global health landscape.

## 2 Methods

### 2.1 Data sources

The data for this study were primarily sourced from the GBD 2021 Study (9). The GBD study is a comprehensive, systematic scientific effort to quantify the comparative health loss from a wide array of diseases, injuries, and risk factors at global, regional, and national levels. It compiles data from a vast number of sources, including population-based surveys, vital registration systems, disease registries, and published literature, providing a rich and globally representative dataset for our analysis of HOA.

### 2.2 Data collection

The data collection process within the GBD study is highly standardized and rigorous. Trained researchers and data collectors from around the world contribute to gathering relevant health information. For HOA-specific data, multiple data collection methods were employed. In population-based surveys, individuals were interviewed about their symptoms, medical history, and any diagnosed joint conditions. Medical records from hospitals, clinics, and primary care providers were also systematically abstracted to identify cases of HOA. Additionally, disease registries, where available, provided valuable longitudinal data on the incidence and prevalence of HOA. The use of multiple data collection methods helps to ensure the comprehensiveness and accuracy of the HOA-related data included in the GBD dataset (10).

### 2.3 Statistical analysis

First, the description of the disease burden was carried out. In 2021, the number of incidence, prevalence, and DALYs of HOA, along with their corresponding age-standardized rates (ASRs), were reported globally and stratified by different sub-types. These sub-types included sex, age groups, SDI regions, GBD regions, and individual countries.

Second, to explore the temporal trend of the disease burden, data from 1990 to 2021 were analyzed both globally and by sub-types. To quantify temporal trends in the ASRs of incidence, prevalence, and DALYs, we estimated the Estimated Annual Percentage Change (EAPC) using a log-linear regression model. ASRs were calculated using the direct standardization method by applying age-specific rates to the GBD 2021 global standard population, which includes 5-year age groups from <1 year to ≥95 years (1). This approach enables comparison across populations by adjusting for differences in age structure. Specifically, we fitted the natural logarithm of the ASR against calendar year as follows:


$$\begin{array}{l} \text{ln}\left(\text{ASR}\right)\;=\text{ α }+\text{ β }\times \text{ year }+\text{ ε} \end{array}$$

where β represents the annual rate of change. The EAPC and its 95% confidence interval (CI) were then calculated as:


$$\begin{array}{l} \text{EAPC }=\;100\;\times \;\left(\text{exp }\left(\text{β}\right)\;-\;1\right) \end{array}$$

A statistically significant increasing trend was defined as an EAPC > 0 with a 95% CI entirely above zero; a decreasing trend as an EAPC <0 with a 95% CI entirely below zero; and a stable trend when the 95% CI included zero. This method allowed us to robustly assess the temporal dynamics of HOA burden across populations.

Based on the obtained EAPC values for age-standardized incidence rate (ASIR), age-standardized prevalence rate (ASPR), and age-standardized DALYs rate (ASDAR), we conducted a hierarchical agglomerative cluster analysis to identify regions with similar temporal patterns of HOA burden. Each of the 50 GBD regions was represented by a three-dimensional vector (EAPC of incidence, prevalence, and DALYs). Clustering was performed using Ward's minimum variance method as the linkage criterion and Euclidean distance as the dissimilarity metric. The optimal number of clusters was selected by visually inspecting the dendrogram structure to ensure both within-cluster homogeneity and between-cluster separation. This approach enabled the identification of epidemiologically similar regions and provided insight into global trend heterogeneity. All geographic classifications, including SDI strata and GBD regions, were based on the GBD 2021 framework to ensure standardization and comparability.

Finally, to predict the future disease burden from 2020 to 2044, the APC model under the maximum likelihood framework was applied. To forecast the future burden of HOA, we employed an APC model under a maximum likelihood framework. The APC model disentangles the effects of age, calendar period, and birth cohort on disease rates, capturing demographic and generational shifts over time. It assumes a relatively stable population structure and risk factor distribution, making it suitable for chronic disease projections. Compared with time-series models, the APC approach offers superior epidemiological interpretability and has been widely validated in disease burden forecasting, including in GBD studies (11, 12). Its application has also shown superior performance over conventional time-series models in chronic disease projections (13).

All analyses, including data management, statistical modeling, and figure generation, were conducted using R software (version 4.0.2; R Foundation for Statistical Computing, Vienna, Austria). Standard errors for estimates were derived via likelihood-based inference. Statistical significance was defined as a two-tailed *P* value < 0.05.

## 3 Results

### 3.1 The disease burden attributable to HOA in 2021

In 2021, the number of HOA-related incidence cases amounted to 10,367,241 [95% uncertainty intervals (UI): 7,686,291–13,251,881]. The corresponding ASIR was 119.09 (95% UI: 88.73–151.13) per 100,000 population. The number of HOA-related prevalence cases reached 194,284,754 (95% UI: 146,479,865–248,177,668) in 2021, with a corresponding ASPR of 2,237.78 (95% UI: 1,693.67–2,851.21) per 100,000 population. The number of DALYs attributable to HOA was 6,167,308 (95% UI: 2,803,411–12,737,380), and the corresponding ASDAR was 70.94 (95% UI: 32.23–146.27) per 100,000 population (Supplementary Tables S1–S3).

In 2021, the number of incidence, prevalence, and DALYs cases in females was 1.83, 2.00, and 1.97 times higher than that in males, respectively. The corresponding ASRs in females were 1.75, 1.78, and 1.76 times those in males (Supplementary Figure S2, Supplementary Tables S1–S3).

The distribution of incidence, prevalence, and DALYs across age groups in 2021 is presented in Supplementary Figure S3. The number of incidence, prevalence, and DALYs cases initially increased with age, reaching a peak and then declining. The ASPR and ASDAR continuously increased with age. However, the ASIR first increased with age, then decreased, and finally increased again (Supplementary Figure S3, Supplementary Tables S1–S3).

At the SDI region level, the middle SDI region had the largest number of incidence cases, which was 3,413,861 (95% UI: 2,523,021–4,379,118), prevalence cases of 57,871,145 (95% UI: 43,457,254–73,924,364), and DALYs cases of 1,843,413 (95% UI: 837,552–3,833,754). The highest ASRs were observed in the high SDI region, with an ASIR of 147.54 (95% UI: 109.86–188.12), an ASPR of 2,780.32 (95% UI: 2,098.02–3,562.49), and an ASDAR of 88.47 (95% UI: 40.04–182.48; Supplementary Figure S4, Supplementary Tables S1–S3).

Across the 50 GBD regions, Asia ranked the top one in number of incidence cases (5,758,148), followed by Advanced Health System (3,395,089) and Basic Health System (4,275,470). Advanced Health System also ranked the top one for number of prevalence cases (78,047,872), followed by Asia (100,372,746) and Europe (42,559,011). For DALYs cases, Asia led (3,197,459), followed by Advanced Health System (2,465,289) and America (1,202,432). However, Oceania ranked the bottom one for incidence (8,831), prevalence (126,572), and DALYs (4,061). For ASRs, Central Asia had the highest ASIR (194.58/100,000), while Minimal Health System ranked the lowest (76.71/100,000). The highest ASPR was in Central Asia (3,903.67/100,000), and the lowest in Minimal Health System (1,387.27/100,000). Central Asia also led in ASDAR (124.56/100,000), with Minimal Health System again at the bottom (43.77/100,000). Regions consistently ranking low included Commonwealth Low Income, Western Africa, and Western Sub-Saharan Africa (Supplementary Figure S5, Supplementary Tables S1–S3).

**Figure 1:**
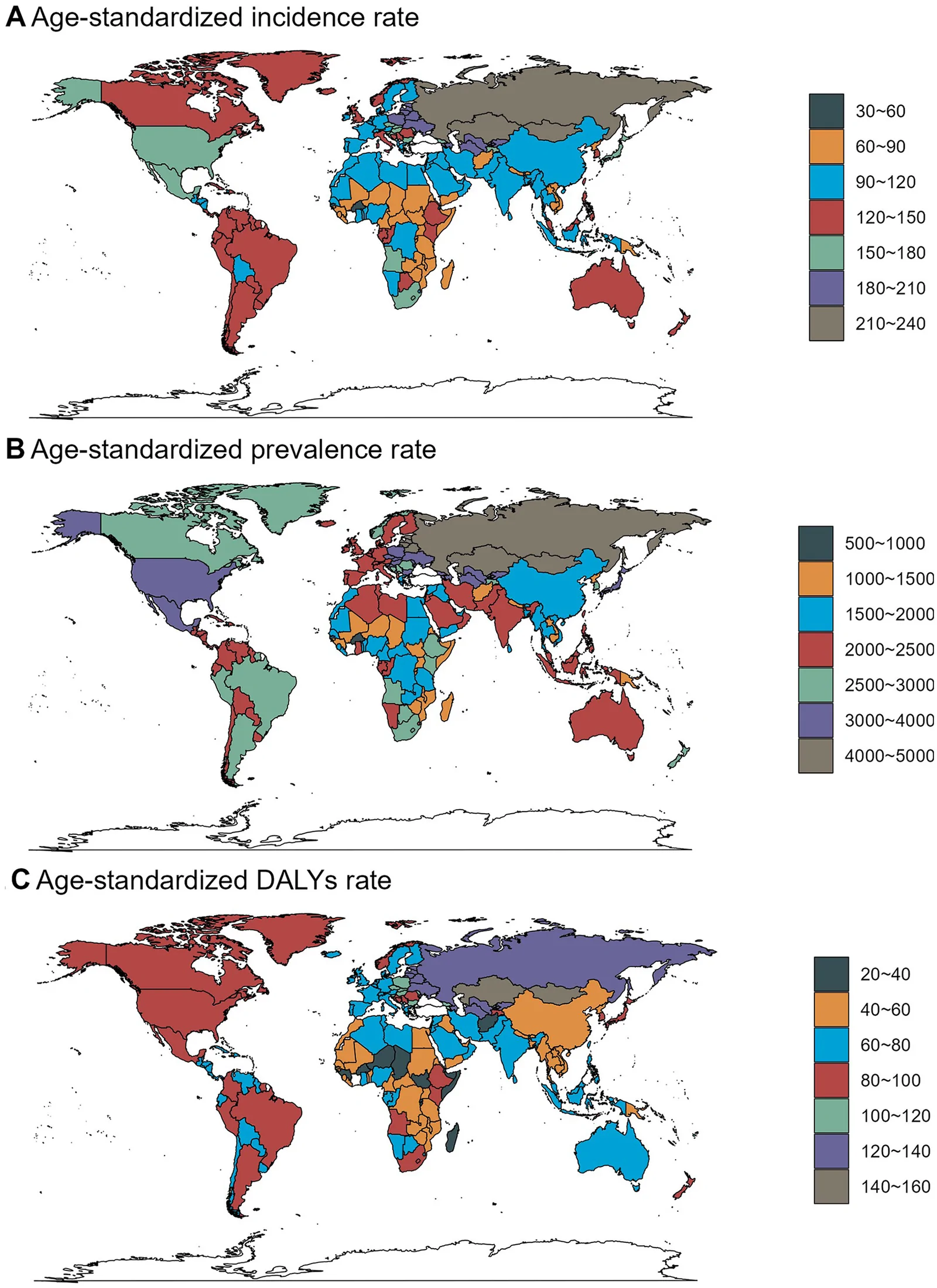
Age-standardized rates of osteoarthritis hand-related incidence, prevalence, and DALYs across countries and territories in 2021.

The disease burden of HOA varied considerably across the world, with the highest ASIR observed in Mongolia (217.97/100,000), followed by Kazakhstan (221.12/100,000) and Estonia (204/100,000). The lowest ASIR was in Burkina Faso (57.2/100,000), followed by South Sudan (61.98/100,000) and Niger (62.09/100,000). For ASPR, the highest rates were in Mongolia (4,430.54/100,000), Latvia (4,083.12/100,000), and Lithuania (4,032.3/100,000), while the lowest were in Burkina Faso (995.97/100,000), Madagascar (1,102.49/100,000), and Burundi (1,244.52/100,000). The highest ASDAR was in Mongolia (141.61/100,000), followed by Kazakhstan (145.11/100,000) and Turkmenistan (123.93/100,000), with the lowest in Burkina Faso (31.69/100,000), South Sudan (33.57/100,000), and Niger (34.31/100,000). In terms of absolute numbers, China had the highest number of incidence cases (1,960,255), followed by India (1,563,214) and the United States of America (803,664). The United States of America also led in number of prevalence (17,926,585) and DALYs (558,646) cases, followed by China (34,420,008 prevalence; 1,103,886 DALYs) and India (25,505,889 prevalence; 801,114 DALYs). The lowest numbers were in Tokelau (two incidence, 35 prevalence, one DALY), followed by Niue (three incidence, 55 prevalence, two DALY) and Nauru (10 incidence, 144 prevalence, five DALY; Figure 1, Supplementary Tables S1–S3, Supplementary Figure S1). Estimates from microstate territories such as Tokelau and Niue should be interpreted with caution due to small population sizes and potential modeling uncertainty.

### 3.2 Temporal trend for HOA-related disease burden from 1990 to 2021

**Figure 2:**
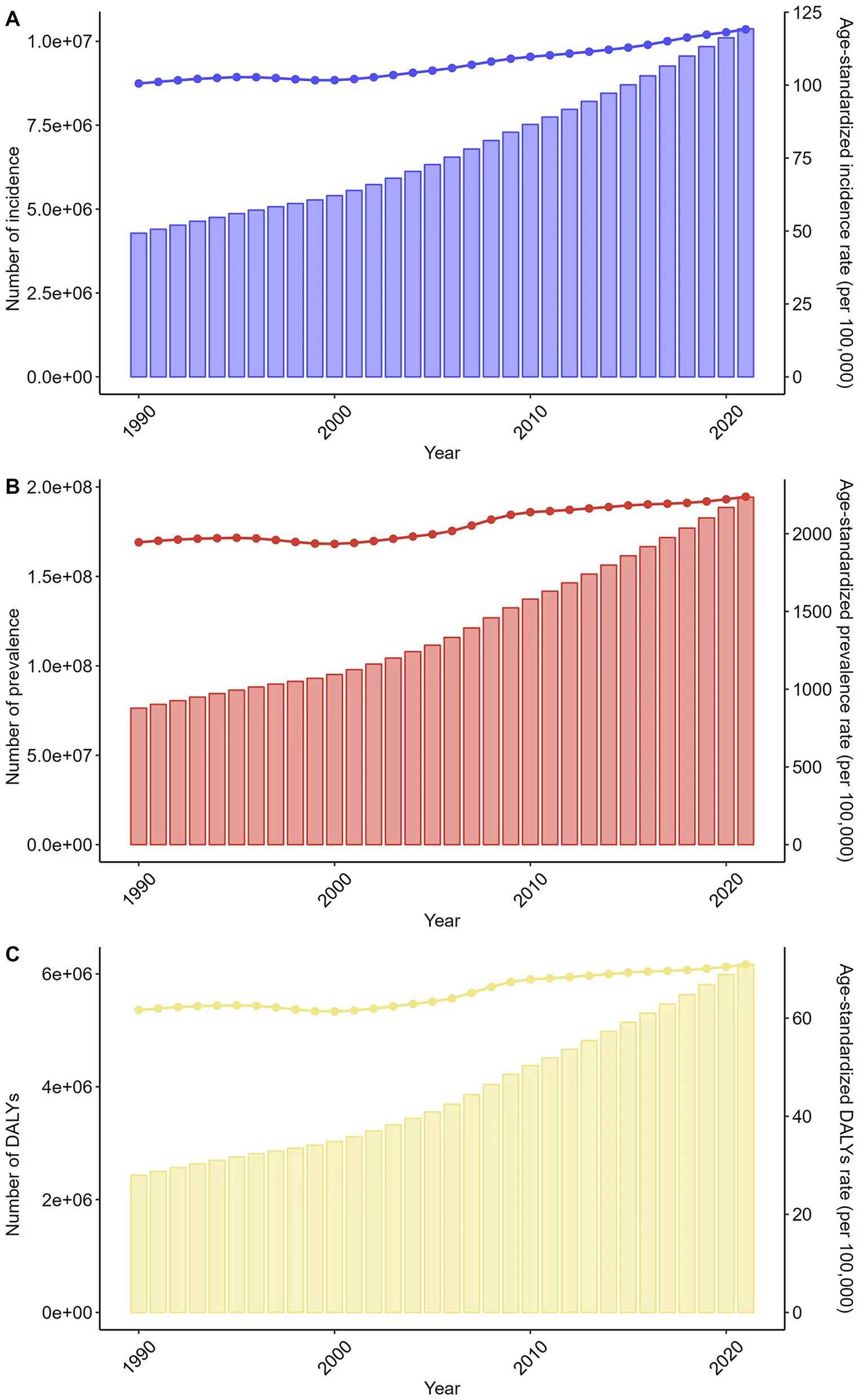
Trends in the numbers and age-standardized rates of osteoarthritis hand-related incidence, prevalence, and DALYs globally from 1990 to 2021.

Globally, the number of HOA incidence cases increased from 4,282,225 (95% UI: 3,174,006–5,467,719) in 1990 to 10,367,241 (95% UI: 7,686,291–13,251,881) in 2021. The number of prevalence cases rose from 76,317,645 (95% UI: 57,748,915–97,654,829) to 194,284,754 (95% UI: 146,479,865–248,177,668), and the number of DALYs cases increased from 2,432,664 (95% UI: 1,103,100–5,033,222) to 6,167,308 (95% UI: 2,803,411–12,737,380). Regarding the corresponding ASRs, they all changed in the same upward direction. The ASIR increased from 100.57 (95% UI: 74.51–128.13) to 119.09 (95% UI: 88.73–151.13), the ASPR increased from 1,944.84 (95% UI: 1,476.85–2,478.43) to 2,237.78 (95% UI: 1,693.67–2,851.21), and the ASDAR increased from 61.67 (95% UI: 27.93–127.04) to 70.94 (95% UI: 32.23–146.27) per 100,000 population (Figure 2, Supplementary Tables S1–S3).

The trends in males and females separately were consistent with those of the overall population (Supplementary Figure S6, Supplementary Tables S1–S3). Additionally, the trends were consistent across all age groups (Supplementary Figure S7, Supplementary Tables S1–S3). At the SDI region level, all SDI regions demonstrated the same trend as the overall population (Supplementary Figure S8, Supplementary Tables S1–S3).

**Figure 3:**
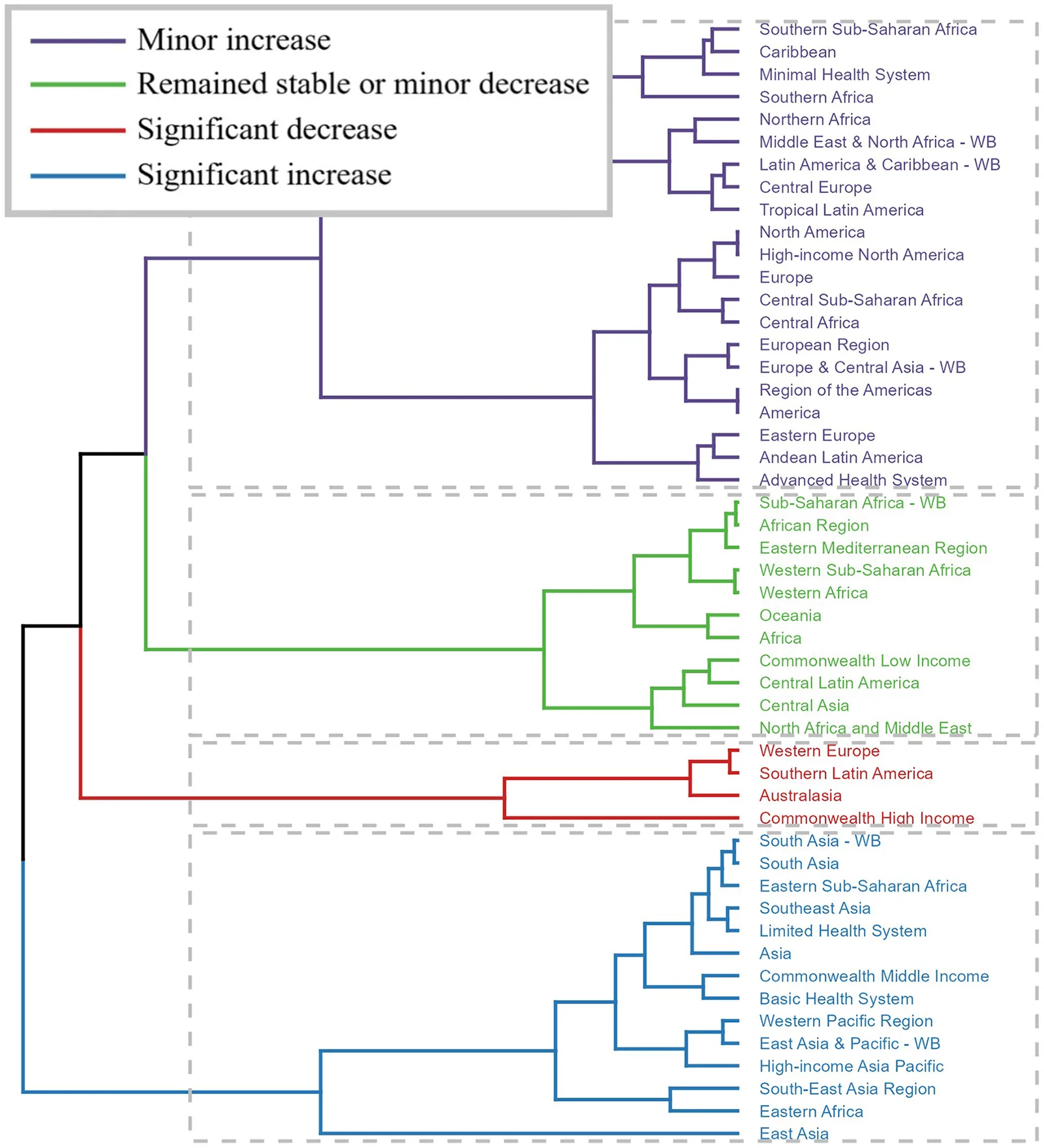
Results of cluster analysis based on the EAPC values of the osteoarthritis hand-related age-standardized rates for incidence, prevalence, and DALYs from 1990 to 2021.

Across GBD regions, the trend of the HOA-related disease burden showed variability. The results of cluster analysis are presented in Figure 3. A significant increase in incidence, prevalence, and DALYs rate occurred in South Asia-WB, South Asia, Eastern Sub-Saharan Africa, Southeast Asia, Limited Health System, Asia, Commonwealth Middle Income, Basic Health System, Western Pacific Region, East Asia & Pacific-WB, High-income Asia Pacific, South-East Asia Region, Eastern Africa, and East Asia. In contrast, a significant decrease was observed in Western Europe, Southern Latin America, Australasia, and Commonwealth High Income (Figure 3).

**Figure 4:**
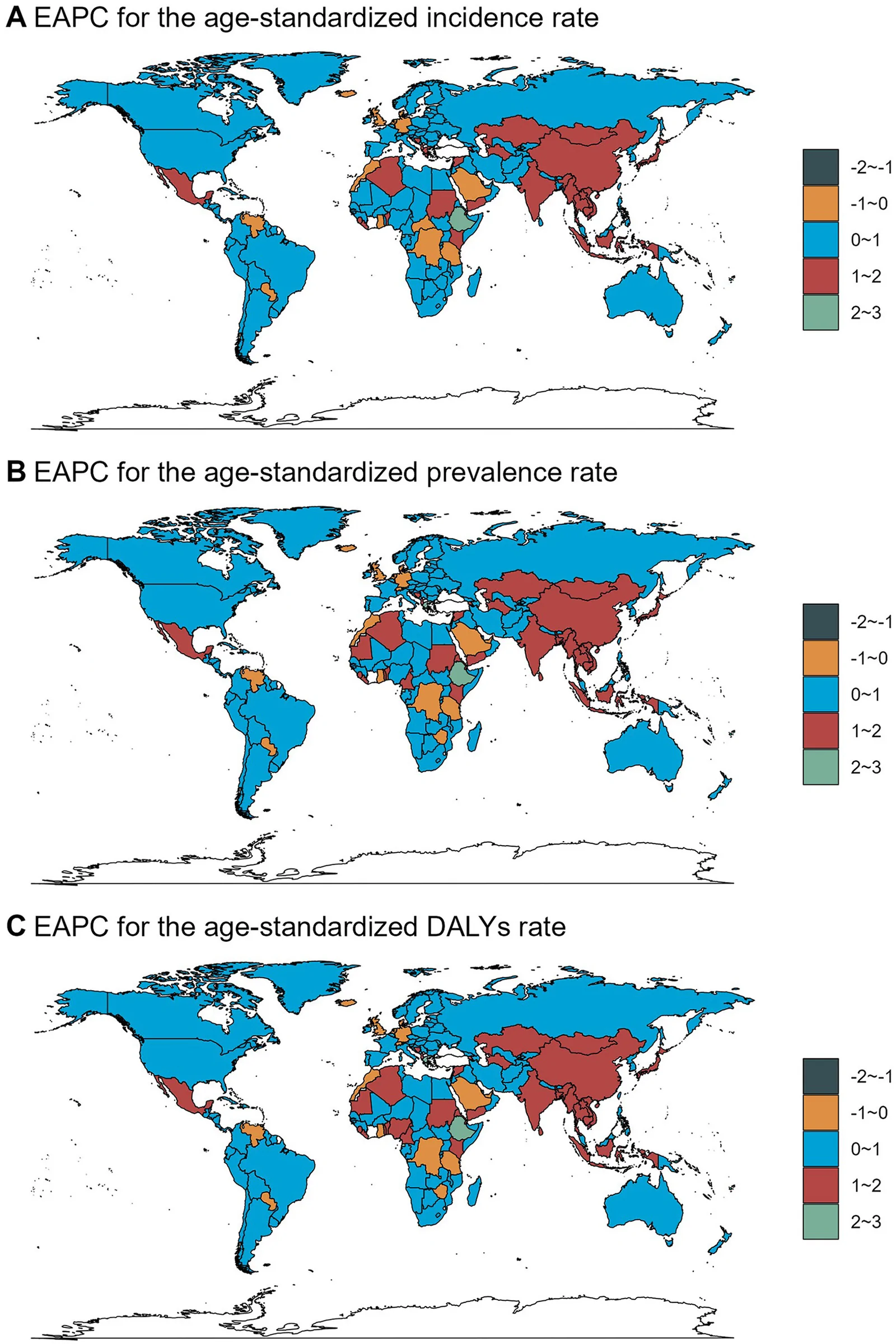
The EAPC of osteoarthritis hand-related ASRs from 1990 to 2021.

Across countries and territories, the changing trend also differed. From 1990 to 2021, Equatorial Guinea exhibited the most significant increase in ASIR [EAPC = 2.53, 95% confidence interval (CI): 2.35–2.7], ASPR (EAPC = 2.77, 95% CI: 2.59–2.95), and ASDAR (EAPC = 2.82, 95% CI: 2.63–3.01). Israel showed the most significant decrease in ASIR (EAPC = −1.33, 95% CI: −1.90 to −0.76), ASPR (EAPC = −1.33, 95% CI: −1.91 to −0.76), and ASDAR (EAPC = −1.36, 95% CI: −1.94 to −0.78; Figure 4, Supplementary Tables S1–S3).

### 3.3 The predicted results from 2022 to 2046

**Figure 5:**
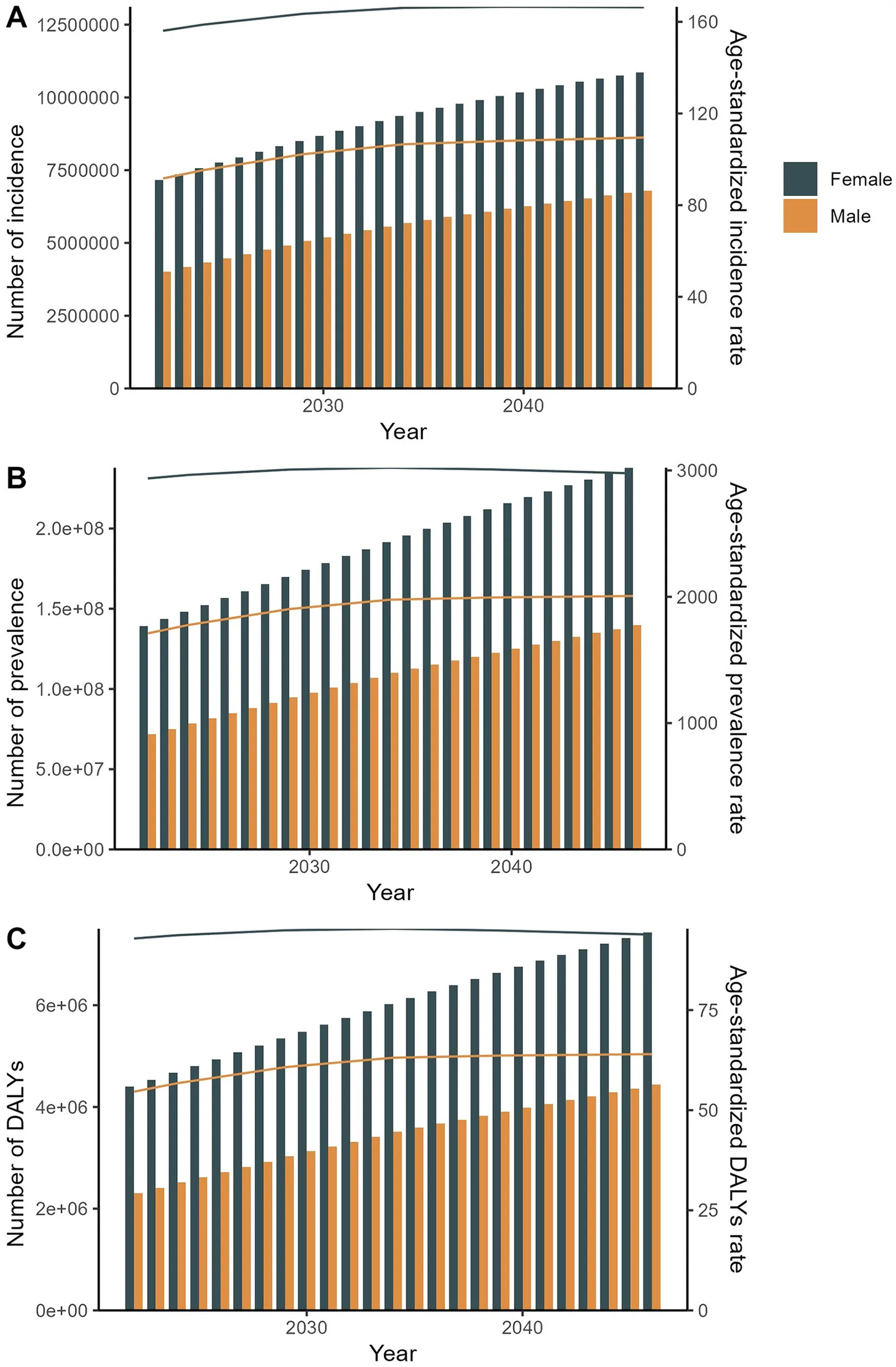
The predicted results in the osteoarthritis hand-related numbers and age-standardized rates of incidence, prevalence, and DALYs by sex globally from 2022 to 2046 of the APC model.

The predicted results of the APC model indicated that the number of incidence, prevalence, and DALYs cases for both genders would increase from 2022 to 2046. For males, the number of incidence cases was 4,010,703 in 2021 and was predicted to be 6,801,339 in 2046. The number of prevalence cases was 71,876,664 in 2022 and was projected to reach 139,709,466 in 2046. The number of DALYs was 2,303,952 in 2022 and was expected to be 4,434,962 in 2046. For females, the number of incidence cases would increase from 7,164,166 in 2022 to 10,864,957 in 2046. The number of prevalence cases would increase from 139,256,250 to 237,872,054 during this period. The number of DALYs cases would increase from 4,399,690 to 7,429,105. Moreover, the predicted results showed that the corresponding ASRs would continue to increase over the next 25 years for both genders. In 2021, the ASIR for males was 91.67 per 100,000 population and was predicted to be 109.50 in 2046. The ASPR for males was 1,710.74 in 2022 and was projected to be 2006.14 in 2046. During the same period, the age-standardized DALYs cases increased from 54.62 to 63.98. For females, the ASIR would increase from 156.10 to 166.37, the ASPR would increase from 2,936.99 to 2,977.00, and the ASDAR would increase from 92.86 to 93.83 (Figure 5, Supplementary Table 4).

## 4 Discussion

### 4.1 Summary of key findings

The present study provides a comprehensive analysis of the global burden of HOA in 2021, temporal trends from 1990 to 2021, and projections through 2046, utilizing data from the GBD study. In 2021, HOA was associated with 10.37 million incidence cases, 194.28 million prevalence cases, and 6.17 million DALYs, with ASRs reflecting substantial global and regional disparities. Females exhibited a significantly higher burden across all metrics, with incidence, prevalence, and DALYs counts 1.83, 2.00, and 1.97 times those of males, respectively, aligning with prior epidemiological evidence of sex-specific susceptibility to OA (14, 15). Age distribution patterns showed a peak in incidence cases at advanced ages, followed by a decline, while prevalence and DALYs continued to rise with age, underscoring the chronic and degenerative nature of HOA (16).

### 4.2 Global and regional disparities

Geographically, middle SDI regions harbored the highest absolute numbers of incidence, prevalence, and DALYs cases, likely driven by large population sizes and increasing aging populations (17). Conversely, high SDI regions reported the highest age-standardized rates, a finding consistent with studies linking higher socioeconomic status to increased life expectancy and age-related OA prevalence, despite better access to healthcare (18). Central Asia emerged as a hotspot for HOA, with the highest ASIR (194.58/100,000), ASPR (3,903.67/100,000), and ASDAR (124.56/100,000), possibly influenced by genetic predispositions, occupational risks, or climate-related factors (19). In contrast, minimal health system regions and sub-Saharan African countries exhibited the lowest rates, which may reflect underdiagnosis, limited healthcare access, or competing disease priorities (20).

Recent studies suggest that this elevated burden in Central Asia may be underpinned by both genetic and occupational determinants. Polymorphisms in the COL1A1 gene, particularly within the Sp1 binding site, are known to compromise collagen structure and have been associated with increased OA susceptibility. These variants have been reported at higher frequencies in Eastern European and Central Asian populations (21). Concurrently, epidemiological data from Kazakhstan and Uzbekistan highlight a disproportionately high prevalence of repetitive manual labor in agricultural and textile sectors, with substantial female representation (22, 23). These mechanistic insights provide biological plausibility for the observed regional ASR patterns and underscore the need for integrative surveillance combining genetic, occupational, and demographic risk stratification.

Community-based arthritis interventions have shown promise in low-resource settings. For instance, India's WHO-supported outreach initiatives have achieved symptom relief and functional improvement through ergonomic education and task modification (24). These scalable, low-cost models offer practical guidance for regions such as Central Asia, where specialized care remains limited. Embedding such strategies within national musculoskeletal health frameworks could strengthen regional responses to the growing HOA burden.

Country-specific analysis highlighted Mongolia, Kazakhstan, and the United States as having high absolute or standardized burdens. The U.S. led in prevalence cases and DALYs, consistent with its aging population and high obesity rates, a known risk factor for OA (25). Conversely, low-burden regions like Tokelau and Niue likely benefit from smaller populations and potentially healthier lifestyles, though data reliability in microstates warrants caution (26).

### 4.3 Temporal trends and projections

From 1990 to 2021, global HOA burdens increased in both absolute and age-standardized terms, with ASIR rising by 18.4% and ASPR by 15.1%. These trends mirror the global rise in non-communicable diseases and align with projections for other OA subtypes, such as knee OA, driven by aging populations and changing lifestyles (27). Cluster analysis revealed divergent regional trends: while low- and middle-income regions (South Asia, sub-Saharan Africa) experienced significant increases, high-income regions like Western Europe and Australasia saw declines, possibly due to improved risk factor management (reduced occupational hazards, better pain management) (28). Equatorial Guinea's steep increases and Israel's declines highlight the role of socioeconomic development, healthcare infrastructure, and public health interventions in shaping disease trajectories (29).

The APC model projections indicate a continued rise in HOA burden through 2046, with female cases expected to outpace male cases due to longer life expectancy and higher baseline prevalence (16). While age-standardized rates for males are projected to increase by 19.4%, females show a more modest 6.6% rise in ASIR, suggesting potential convergence in sex disparities or evolving risk factors (30).

The projected narrowing of sex disparities in ASIR may reflect complex demographic and occupational dynamics. As male life expectancy improves globally—particularly in middle-income countries—the gap in age-related risk exposure between sexes is diminishing (31). Simultaneously, industrial and informal sectors with high physical demand, such as construction and delivery services, are seeing increased male labor participation, potentially intensifying cumulative hand strain (32). In contrast, widespread awareness of musculoskeletal health among women, as well as earlier diagnosis and intervention, may temper further ASIR growth. These opposing trends warrant continued monitoring to evaluate whether the projected convergence reflects structural shifts or transitional dynamics.

The observed heterogeneity in HOA burden trends across regions likely reflects differences in public health infrastructure, aging policies, and occupational safety enforcement. In high-income regions such as Western Europe, declining ASRs may be attributable to longstanding investments in musculoskeletal health promotion, early intervention strategies, and ergonomic workplace adaptations (33). In contrast, rapid increases in South Asia and sub-Saharan Africa may stem from limited access to primary care, low public awareness of joint disorders, and minimal regulatory oversight of occupational hazards (34). Moreover, the absence of robust national strategies for obesity and chronic disease prevention in many low- and middle-SDI countries has likely contributed to the upward trajectory of HOA burden (35, 36). These findings highlight the importance of integrated, multisectoral policies—including weight management programs, aging-friendly workforce initiatives, and improved diagnostic access—to mitigate the growing burden of HOA in vulnerable populations (37).

### 4.4 Comparison with existing literature

Our findings corroborate previous GBD analyses on musculoskeletal disorders, which identify OA as a leading cause of disability worldwide (38). The marked predominance of HOA burden among women is underpinned by intersecting biological and occupational determinants. Endogenously, estrogen plays a critical role in preserving joint integrity by modulating chondrocyte function, proteoglycan synthesis, and inflammatory pathways. The abrupt decline in estrogen following menopause accelerates articular cartilage degeneration and heightens synovial inflammation, rendering women biologically more susceptible to degenerative joint pathology. Meta-analytical evidence corroborates this association, linking post-menopausal estrogen deficiency with the onset and progression of osteoarthritis in both weight-bearing and non-weight-bearing joints, including the hands (13, 39, 40). Although hormone replacement therapy has been proposed as a disease-modifying approach, current evidence remains inconclusive regarding its effectiveness in attenuating HOA progression (41, 42). These pathophysiological mechanisms highlight the fundamental role of hormonal milieu in shaping sex-specific disparities in HOA risk.

Superimposed on this biological vulnerability are occupational exposures that disproportionately affect women. Across many settings, women are overrepresented in informal and underregulated labor sectors—such as caregiving, subsistence agriculture, and textile manufacturing—that involve repetitive fine motor activity and lack ergonomic safeguards (43, 44). These cumulative occupational insults, exacerbated by hormonal vulnerability and gendered health-seeking behavior—characterized by greater symptom reporting and healthcare utilization among women—may increase diagnosis rates (45). However, these trends are often offset by systemic inequities, including delayed referral pathways and implicit clinical biases that may downplay musculoskeletal complaints in women. Together, these factors point to a multifactorial origin of the sex gap in HOA burden, underscoring the imperative for integrated strategies that address both biological and structural drivers through sex-specific prevention, occupational safeguards, and equitable musculoskeletal care delivery.

The inverse U-shaped distribution of HOA incidence—characterized by a peak in middle to late adulthood followed by a subsequent decline—stands in contrast to the progressive, age-dependent increase typically observed in knee osteoarthritis (46). This divergence suggests distinct pathophysiological mechanisms shaped by joint-specific anatomical and functional demands. In the hands, the cumulative impact of fine-motor, repetitive activities performed during working age may drive an earlier onset of joint damage, whereas in weight-bearing joints such as the knee, mechanical load accumulates more linearly with age.

Emerging histological and imaging evidence further supports this divergence (47, 48). Articular cartilage in hand joints exhibits lower proteoglycan content, reduced regenerative capacity, and a heightened pro-inflammatory microenvironment compared with the knee. These tissue-level differences may predispose the hands to earlier mechanical fatigue and accelerated degeneration. Moreover, microtrauma resulting from repetitive precision tasks induces periarticular inflammation via distinct biomechanical pathways that differ from the axial loading forces implicated in knee OA pathogenesis. These findings underscore the importance of considering joint-specific exposures and structural characteristics when interpreting epidemiological patterns and designing targeted preventive strategies.

### 4.5 Study limitations

Several limitations should be acknowledged. First, the use of GBD data assumes consistent diagnostic definitions of HOA across regions, which may lead to underreporting in settings with limited clinical capacity. Second, the absence of structural determinants—such as income inequality, labor informality, and healthcare access—limits interpretation of regional disparities. Third, GBD data lack details on modifiable risks such as occupation or hand trauma, limiting causal inference. Finally, the exclusion of patient-reported outcomes means the lived experience of pain and disability is only partially reflected in DALY estimates.

## 5 Conclusion

This study underscores the substantial and growing global burden of HOA, with pronounced sex, age, and regional disparities. The upward trends in both absolute and standardized rates highlight the need for targeted public health strategies, particularly in high-burden regions and aging populations. While challenges in data accuracy and model assumptions exist, the findings provide a robust evidence base for prioritizing HOA in musculoskeletal health agendas, promoting early intervention, and improving healthcare access. Future research should focus on integrating risk factor data and evaluating the effectiveness of preventive measures to mitigate the projected increase in HOA burden.

## Data Availability

The original contributions presented in the study are included in the article/Supplementary material, further inquiries can be directed to the corresponding authors.
