# Supplementary figures and images for: The global burden of osteoarthritis hand: lessons from the Global Burden of Disease Study 1990–2021
Source: Front Med (Lausanne). 2025 Jul 31;12:1616132. doi: 10.3389/fmed.2025.1616132 (PMC12350485; doi:10.3389/fmed.2025.1616132)

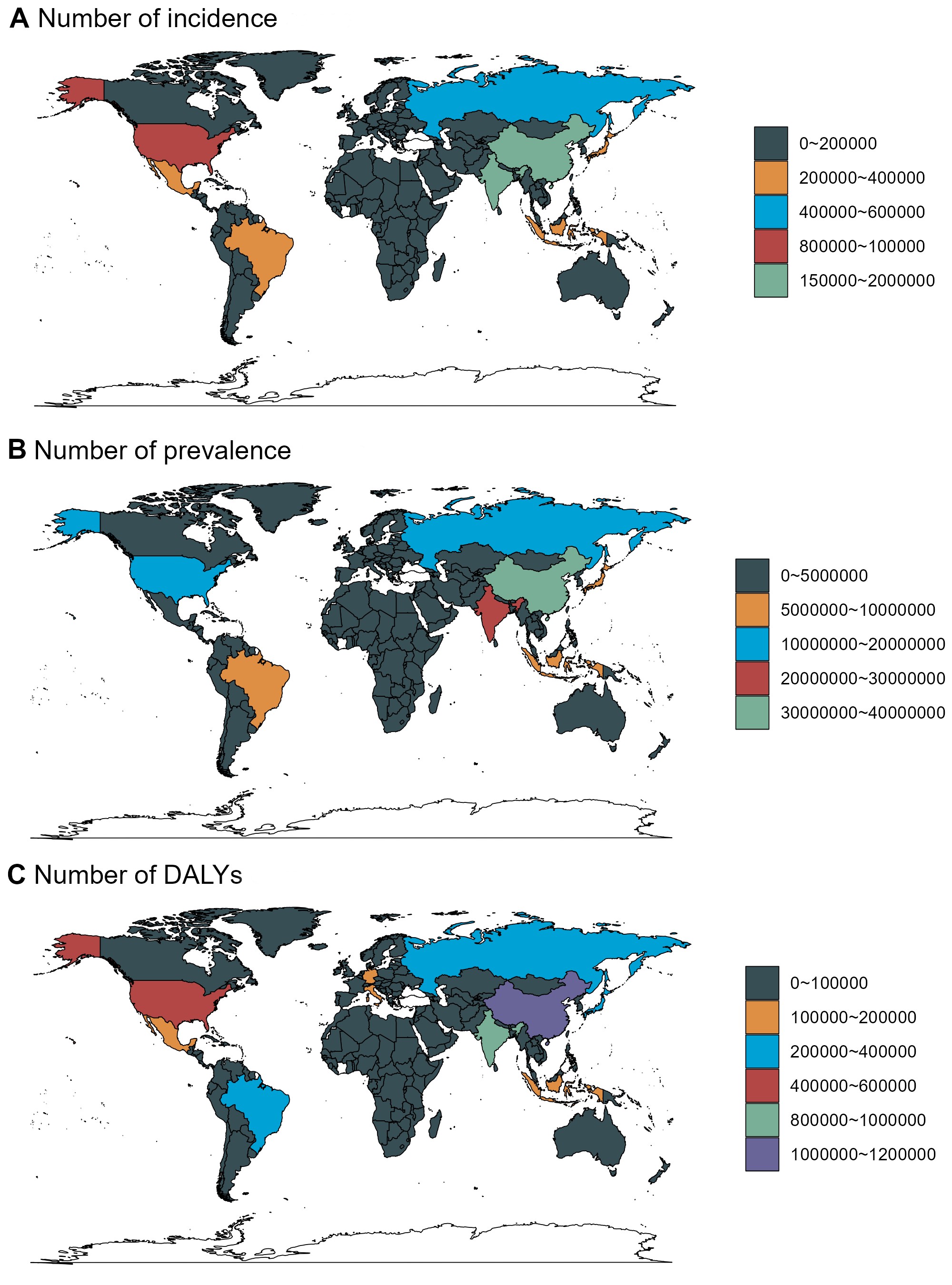

Supplement: Supplementary file 5 [file Image_1.JPEG]

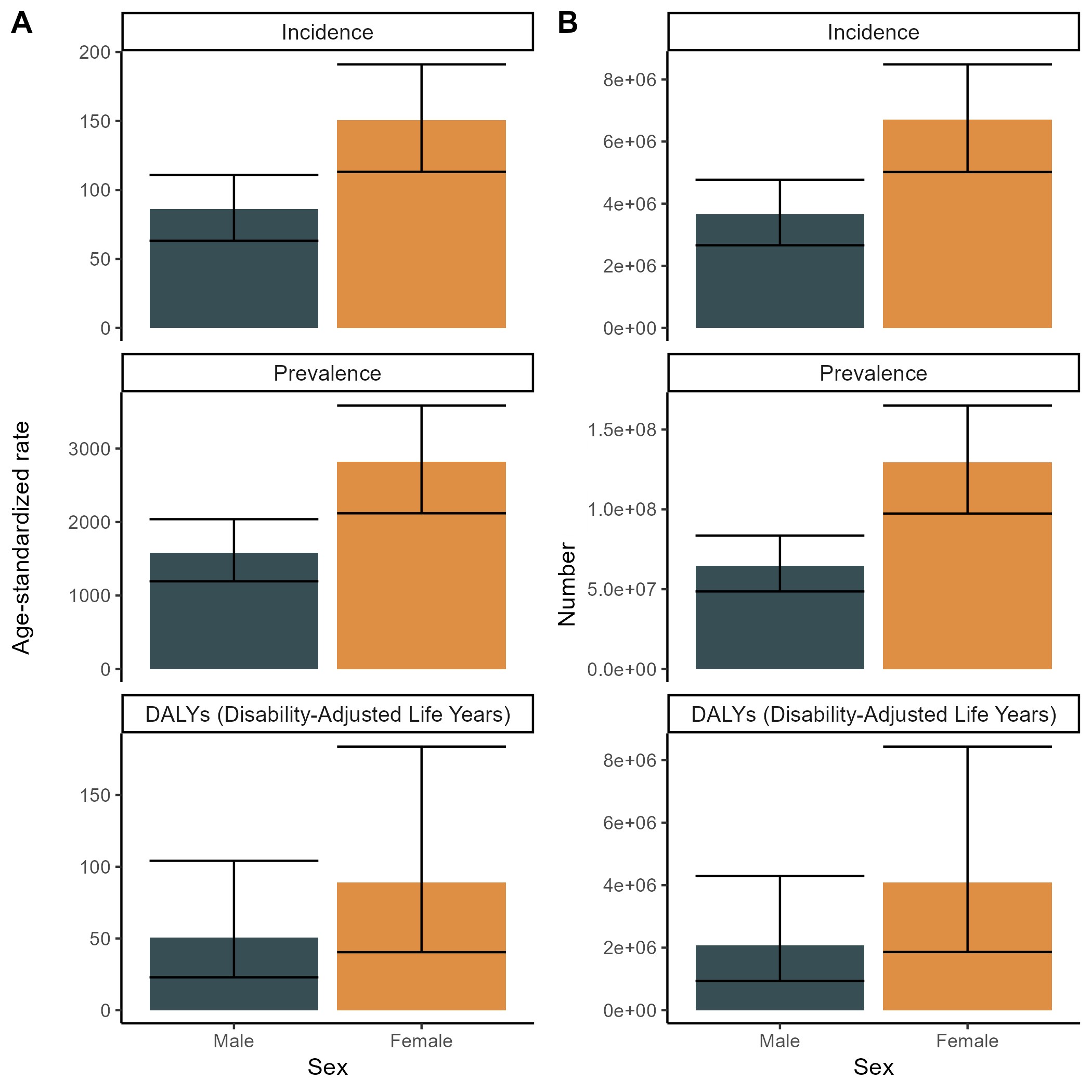

Supplement: Supplementary file 6 [file Image_2.JPEG]

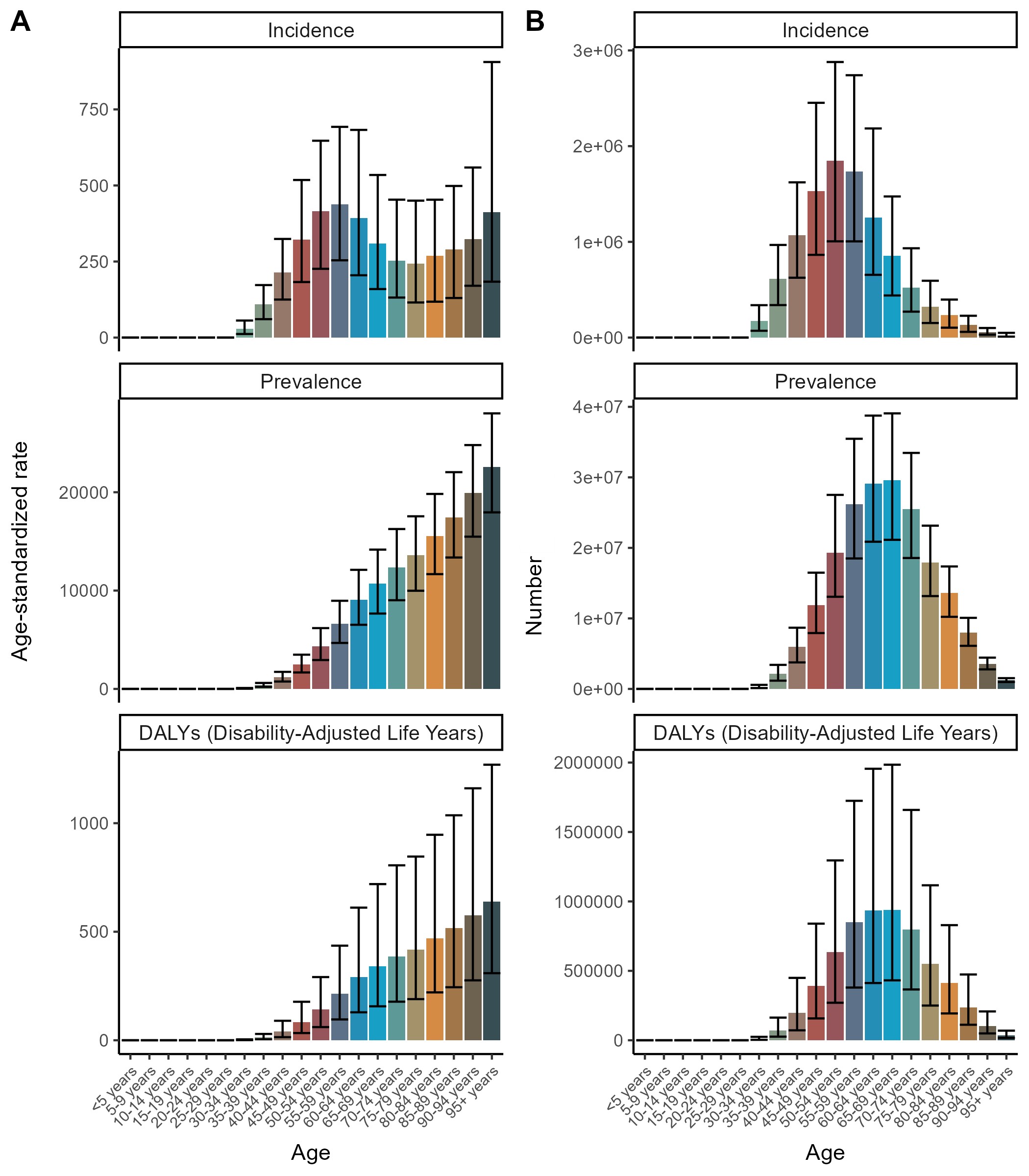

Supplement: Supplementary file 7 [file Image_3.JPEG]

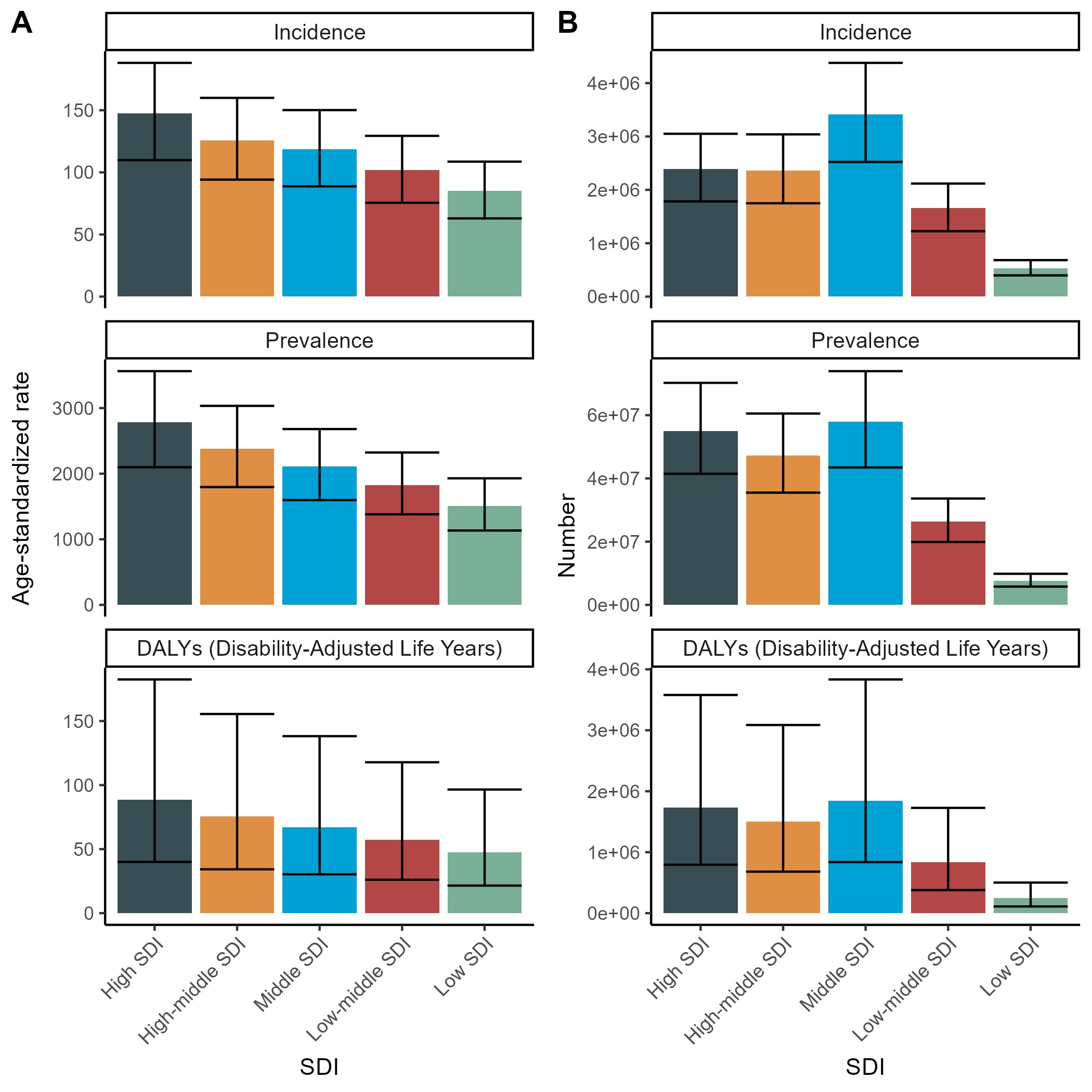

Supplement: Supplementary file 8 [file Image_4.JPEG]

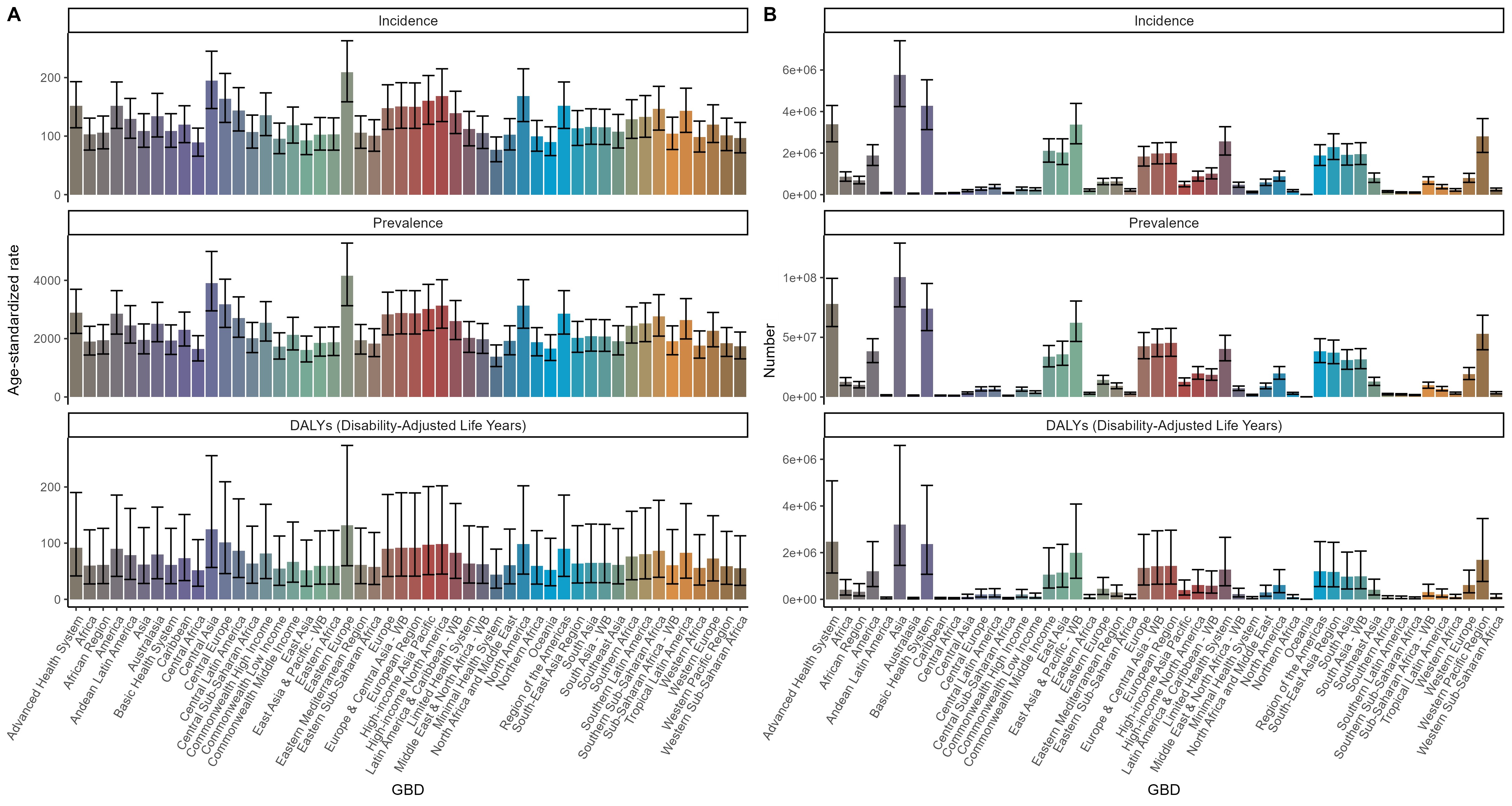

Supplement: Supplementary file 9 [file Image_5.JPEG]

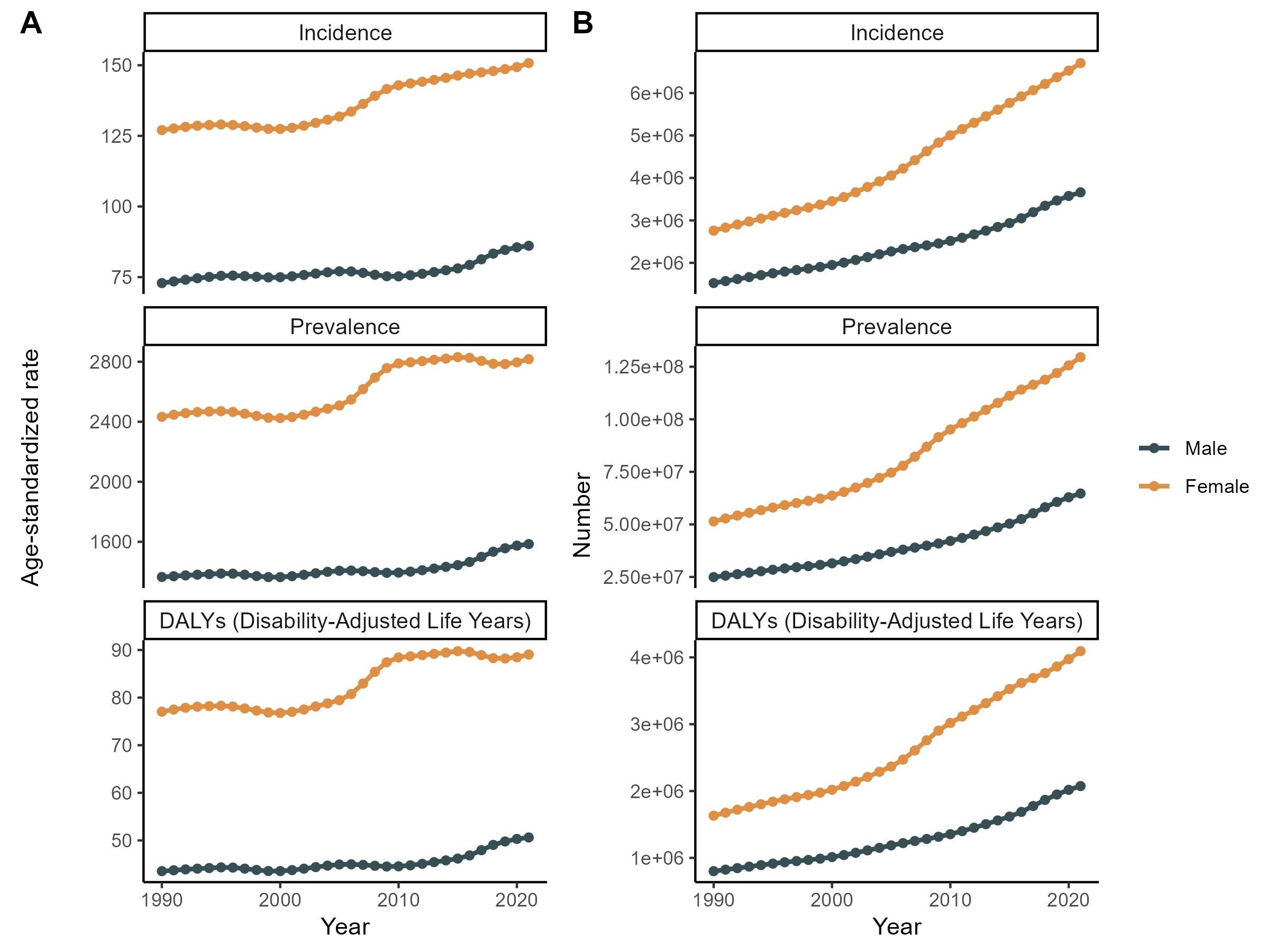

Supplement: Supplementary file 10 [file Image_6.JPEG]

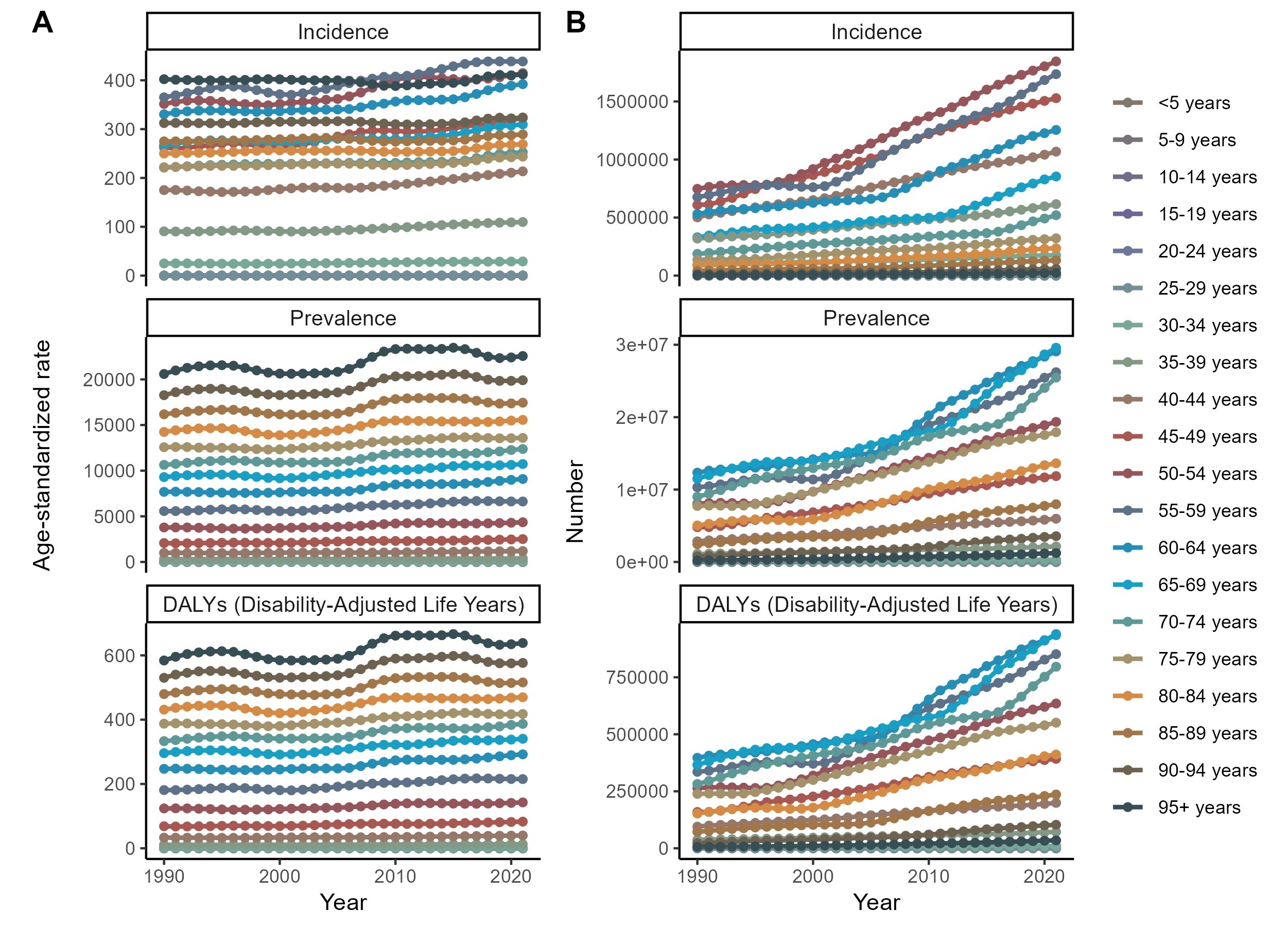

Supplement: Supplementary file 11 [file Image_7.JPEG]

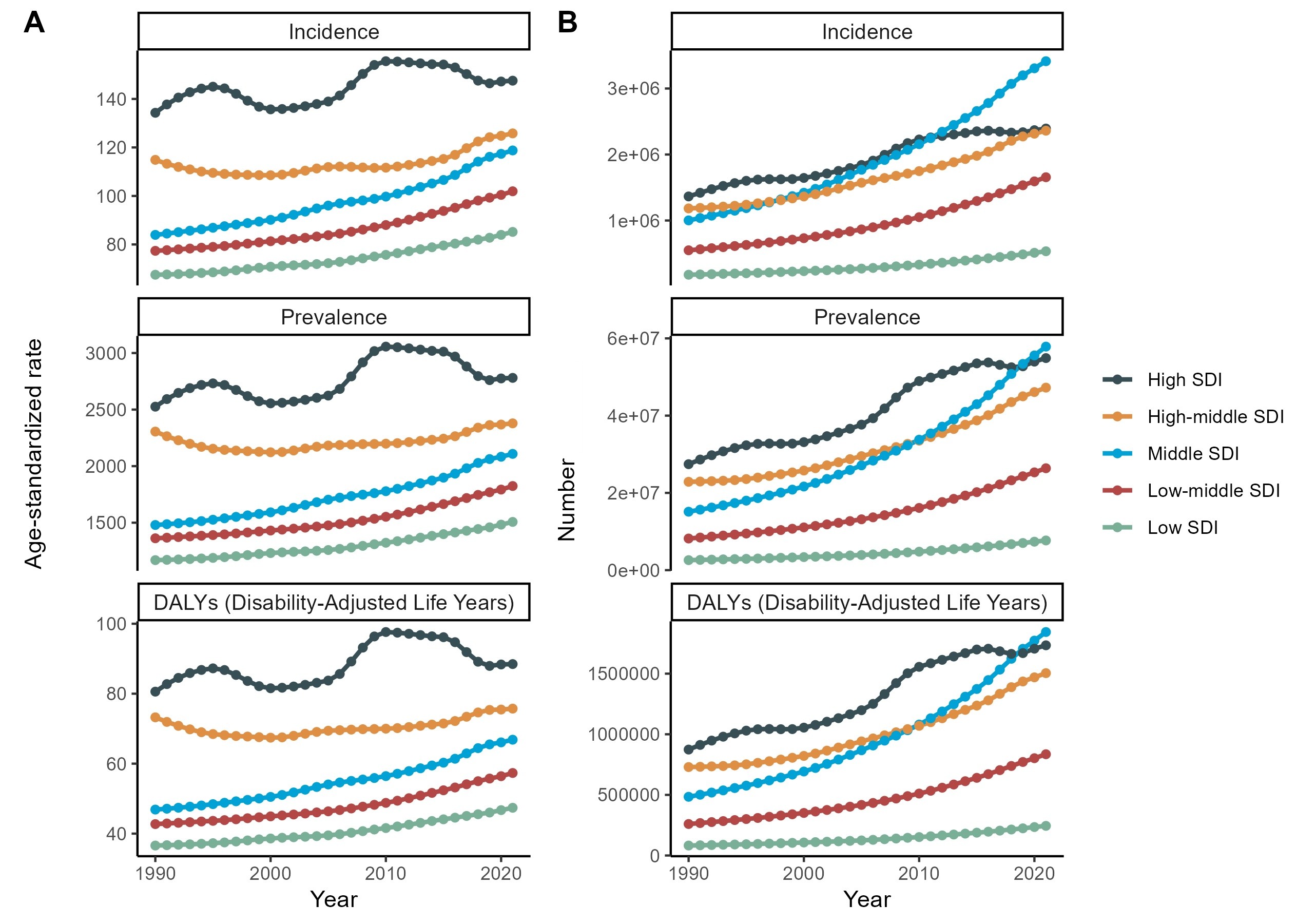

Supplement: Supplementary file 12 [file Image_8.JPEG]
